# Lymphangioma of the ileum causing acute abdominal pain in an adult, a very rare finding during surgery; Case report with literature review

**DOI:** 10.1016/j.ijscr.2019.12.031

**Published:** 2019-12-28

**Authors:** Ayad Ahmad Mohammed, Dildar Haji Musa

**Affiliations:** Department of Surgery, College of Medicine, University of Duhok, Kurdistan Region, Iraq

**Keywords:** Lymphangioma, Gastrointestinal tract, Lymphatic channels, Mesenteric cysts, Abdominal pain

## Abstract

**Introduction:**

Lymphangioma of the gastrointestinal tract is very rare, 75 % of the lesions affect the neck and 20 % affect the axillary region, the rest are seen in the mediastinum, the visceral organs, the retroperitoneum, the mesentery, or in the bones. Lymphangioma of the gastrointestinal tract comprises less than 1 %. The majority of cases are asymptomatic but some cases presented with emergency presentations.

**Case presentation:**

A 31-year-old male presented to emergency department complaining of central abdominal pain for 2 days, with nausea, dyspnea but no vomiting.

The abdominal examination revealed a mildly distended abdomen, with guarding and tenderness mainly in the right lower abdominal quadrant. There were no any palpable masses or organ enlargement. The white blood cells count was elevated (14,000 c/mm).

Abdominal ultrasound showed an evidence of 10*7 cm multiloculated thick walled lesion in the right lower abdomen, suggesting the possibility of an appendicular abscess. The patient was admitted for 2 days with little clinical improvement. Therefore, a laparotomy was performed at which there were 2 yellowish cystic lesions 60 cm from the ileocecal valve that were arising from each side of the small bowel and related to its mesentery causing luminal narrowing. Resection was done with end-end anastomosis. The histopathology results revealed a diagnosis of ill lymphangioma of the ileum.

**Conclusion:**

Complete surgical resection is the main form of therapy whenever possible, sometimes is impossible when there is local invasion. When complete resection is performed long term follow up is not recommended as the recurrence is very rare.

## Highlights

• Lymphangioma of the gastrointestinal tract is very rare, 75 % of the lesions affect the neck and 20 % affect the axillary region.
• The majority of cases are asymptomatic but some cases presented with emergency presentations.
• Complete surgical resection is the main form of therapy whenever possible.

## 1 Introduction

Cystic lymphangioma was first described in 1913 by Gaudier and Gorse, when they define it as rare benign cystic lesions containing chylous or serous fluid and composing of lymphatic channels lined by endothelial cells. In literature it is reported most lesions are diagnosed in children and there is no sex predominance, about 75 % of the lesions affect the neck and 20 % affect the axillary region, the rest of the lesions are seen in some very rare locations such as the mediastinum, the visceral organs, the retroperitoneum, the mesentery, or in the bones. Lymphangioma of the gastrointestinal tract comprises less than 1 % [1,2].

Lymphangioma of the bowel usually arises from the lymphatic channels where the lacteals of the villi empty, lesions are thought to be hamartomatous lesions rather than tumors which enlarges gradually with time. Lymphangiomas are usually solitary and not connected to the lymphatic channels in that region resulting in stasis and proximal dilatation [3].

The majority of cases are asymptomatic and are diagnosed accidentally, most patients present with painless palpable soft abdominal mass, which grows slowly, but in some cases it may cause acute abdominal pain or intestinal obstruction which may be caused by luminal obstruction, or cases reported to be caused by small bowel volvulus, in some rare occasions it may be caused by the traction effect of the mass over the bowel [2,4,5].

The characteristic findings on abdominal CT scan is the presence of oval submucosal well defined cystic lesions which show no enhancement when contrast is injected [1].

The work of this report case has been reported in line with the SCARE 2018 criteria [6].

## 2 Patient information

A 31-year-old male, who is an office worker presented to emergency department complaining of central abdominal pain for 2 days. d with nausea, dyspnea but no vomiting.

The patients had many attacks of similar pain but of milder intensity for the last years which was diagnosed as irritable bowel syndrome, the surgical histories were unremarkable and the family history was negative for chronic illnesses and malignancies.

### 2.1 Clinical findings

The patient had tachycardia (the pulse rate was 110 b/m) with normal temperature and blood pressure. Other parts of the general examination were normal.

The abdominal examination revealed a mildly distended abdomen, with guarding and tenderness mainly in the right lower abdominal quadrant. There were no any palpable masses or organ enlargement. The bowel sounds were normal.

### 2.2 Diagnostic assessment

The white blood cells count was elevated (14,000 c/mm) and the hemoglobin and the urinalysis were normal.

**Fig. 1:**
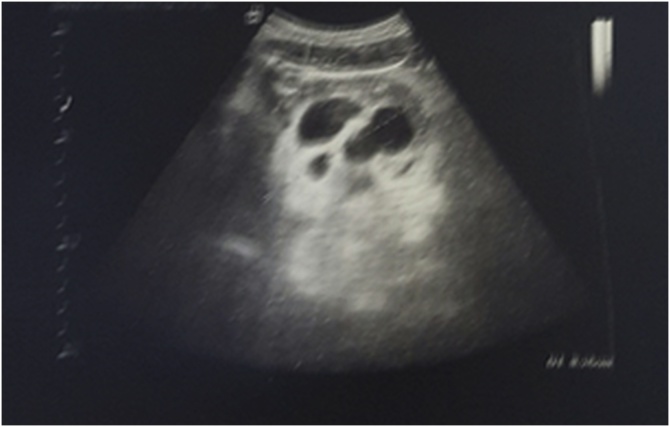
An ultrasound picture showing a 10*7 cm multiloculated thick walled lesion.

Abdominal ultrasound showed an evidence of 10*7 cm multiloculated thick walled lesion in the right lower abdomen which was located lateral to the iliac vessels, suggesting the possibility of an appendicular abscess Fig. 1.

The patient was admitted for 2 days and he received broad spectrum parenteral antibiotics with little clinical improvement.

### 2.3 Therapeutic intervention

**Fig. 2:**
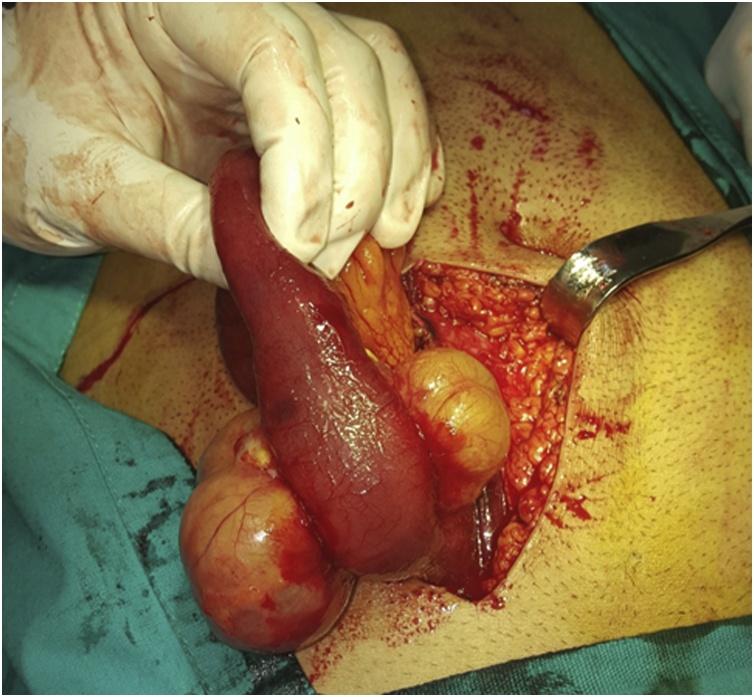
An intraoperative picture showing 2 yellowish cystic lesions related to the terminal ileum and causing luminal narrowing.

**Fig. 3:**
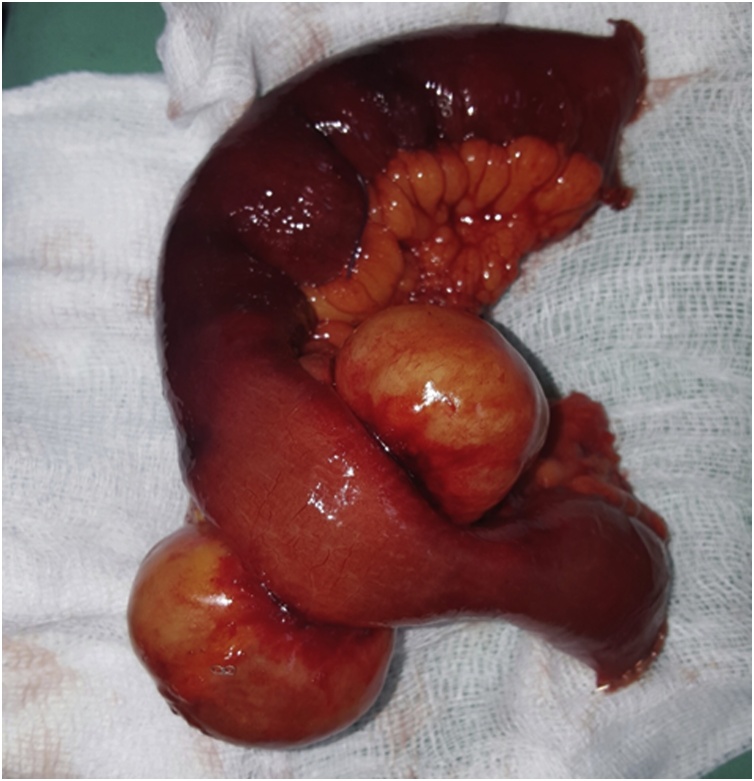
An intraoperative picture showing the lymphangioma after being resected with a segment of healthy looking bowel.

The decision was done for surgery. Therefore, a laparotomy was performed at which there were 2 yellowish cystic lesions that were arising from each side of the small bowel and related to its mesentery causing luminal narrowing. The lesion was located approximately 60 cm from the ileocecal valve. Resection of the affected bowel segment was done with end-end anastomosis by a slowly absorbable suture material. The other parts of the bowel were examined and there were no other pathologies detected Figs. 2 & 3 .

The resected sample was sent for the histopathological examination which showed an evidence of numerous and variable sized lymphatic channels in the mucosa and the submucosa of the bowel, there was intense inflammatory cell infiltration and the sample was negative for malignant cells. The diagnosis was lymphangioma of the ileum.

### 2.4 Follow-up and outcomes

The patient was well in the post-operative period and he was discharged home after 5 days with no post-operative events.

## 3 Discussion

The majority of the lesions are diagnosed incidentally, but rarely the condition is diagnosed before surgery. The disease may be suspected with the typical radiological features, sometimes when endoscopy or enteroscopy is performed the lesions appear as elevated, yellowish or white submucosal polypoidal lesions with intact overlying mucosa, there is dilatation of the mucosal blood vessels over the affected part [1,7].

Lymphangiomas occur due to congenital malformation of the lymphatic channels, it is very important to differentiate these lesions from lymphangiectasia which is defined as dilatation of a previously normal lymphatic channels which is caused by lymphatic obstruction from various inflammatory or malignant conditions, histologically lymphangiectasia also lacks the endothelial lining and the investing smooth muscle components [8].

In the pediatric age group, lymphangiomas tend to be larger in size than those in adults, and most arise from the mesentery, and clinically most patients have emergency presentation such as acute abdomen or intestinal obstruction, while in adults they usually discovered incidentally or present with abdominal mass [8].

Intra-abdominal lymphangiomas usually occur in the retroperitoneum, but may affect the small bowel mesentery, liver and the pancreas. Lesions are usually confused with mesenteric cysts which are of mesothelial origin, lymphangiomas should be differentiated from mesenteric cyst because they have more aggressive behavior and tend to be locally destructive lesions with higher recurrence rate, other differential diagnoses may include congenital duplications cysts of the bowel, cystic tumors, and parasitic cysts especially in endemic regions of hydatid disease [[9], [10], [11]].

Most cases are treated surgically, but the lesions tend to be diagnosed late when they become large in size due to the asymptomatic pattern of the disease. When the lesions cause complications they present earlier. Small lesions less than 2 cm may be managed endoscopically by submucosal resection, but this is done only for lesions which are accessible endoscopically [12].

Complete surgical resection is the main form of therapy whenever possible, sometimes resection with normal surrounding tissue. Complete surgical resection sometimes is impossible when there is local invasion [9].

When complete resection is done long term follow up is not recommended as the recurrence is very rare, but when the complete resection is not possible close follow up is recommended [9].

### 3.1 Patient’s perspective

I was told before surgery that my appendix is perforated and I need an emergency surgery, after surgery I have been told that a part of my bowel was resected and will have no major concerns in the future.

## Conflicts of interest

The author has no conflicts of interest to declare.

## Sources of funding

None.

## Ethical approval

Ethical approval has been exempted by my institution for reporting this case.

## Consent

Written informed consent was obtained from the patient for publication of this case report and accompanying images.

## Author contribution

The concept of reporting the case, data recording, and drafting the work done by Dr Dildar Haji Musa and Dr Ayad Ahmad Mohammed.

Dr Dildar Haji Musa took the consent from the patient for publishing the case.

Final approval of the work to be published was done by Dr Ayad Ahmad Mohammed.

## Registration of research studies

This work is case report and there is no need of registration.

## Guarantor

Dr Ayad Ahmad Mohammed is guarantor for the work.

## Provenance and peer review

Not commissioned, externally peer-reviewed.
